# Association between Dietary Intake and Coronary Artery Calcification in Non-Dialysis Chronic Kidney Disease: The PROGREDIR Study

**DOI:** 10.3390/nu10030372

**Published:** 2018-03-19

**Authors:** Alisson Diego Machado, Luz Marina Gómez, Dirce Maria Lobo Marchioni, Fernanda Silva Nogueira dos Anjos, Maria del Carmen Bisi Molina, Paulo Andrade Lotufo, Isabela Judith Martins Benseñor, Silvia Maria de Oliveira Titan

**Affiliations:** 1 Department of Nephrology, Hospital das Clínicas, Faculty of Medicine, University of São Paulo, São Paulo SP 05403-000, Brazil; fernandanogueiraanjos@hotmail.com (F.S.N.d.A.); smotitan@gmail.com (S.M.d.O.T.); 2 Heart Institute, Hospital das Clínicas, Faculty of Medicine, University of São Paulo, São Paulo SP 05403-900, Brazil; lgomez928@gmail.com; 3 Department of Nutrition, School of Public Health, University of São Paulo, São Paulo SP 03178-200, Brazil; marchioni@usp.br; 4 Health Sciences Center, Federal University of Espírito Santo, Vitória ES 29043-900, Brazil; maria.molina@ufes.br; 5 General Medicine Unit, Faculty of Medicine, and Hospital Universitário, University of São Paulo, São Paulo SP 05508-000, Brazil; palotufo@usp.br (P.A.L.); isabensenor@gmail.com (I.J.M.B.)

**Keywords:** vascular calcification, diet, micronutrients, phosphorus, calcium, potassium, renal insufficiency, chronic, health surveys

## Abstract

Coronary artery calcification (CAC) is a widespread condition in chronic kidney disease (CKD). Diet may play an important role in CAC, but this role is not clear. This study evaluated the association between macro-and micronutrient intakes and CAC in non-dialysis CKD patients. We analyzed the baseline data from 454 participants of the PROGREDIR study. Dietary intake was evaluated by a food frequency questionnaire. CAC was measured by computed tomography. After exclusion of participants with a coronary stent, 373 people remained for the analyses. The highest tertile of CAC was directly associated with the intake of phosphorus, calcium and magnesium. There was a higher intake of pantothenic acid and potassium in the second tertile. After adjustments for confounding variables, the intake of pantothenic acid, phosphorus, calcium and potassium remained associated with CAC in the generalized linear mixed models. In order to handle the collinearity between these nutrients, we used the LASSO (least absolute shrinkage and selection operator) regression to evaluate the nutrients associated with CAC variability. In this approach, the nutrients that most explained the variance of CAC were phosphorus, calcium and potassium. Prospective studies are needed to confirm these findings and assess the role of interventions regarding these micronutrients on CAC prevention and progression.

## 1. Introduction

Vascular calcification (VC) is a widespread and intense condition related to chronic kidney disease (CKD). While in people with normal kidney function and in those with high cardiovascular risk, the prevalence of VC is 20% and 35%, respectively [1,2], it reaches a prevalence of 45% to 70% in non-dialysis CKD patients and up to 90% in dialysis patients [3,4,5,6]. More importantly, VC is associated with a very high risk of mortality, cardiovascular events and other comorbidities [7,8].

VC physiopathology is complex, particularly in CKD, and has multiple contributing factors. Diabetes, insulin resistance and hypertension, which are risk factors for VC, are also very common in CKD. In addition, renal failure adds new mechanisms possibly related to VC, such as an increase in fibroblast growth factor 23 (FGF23) and parathyroid hormone (PTH), hyperphosphatemia, and reduced levels of vitamin D, Klotho and calcification inhibitors, such as pyrophosphate, fetuin-A and matrix Gla protein [9,10,11,12,13,14].

Besides these factors, diet may play an important role in VC, particularly in CKD, and is a potential target for therapeutic measures. Experimental studies have shown the roles of high phosphorus and calcium diets as promoters of calcification, and magnesium and vitamin K intakes as inhibitory factors [15,16,17,18]. However, results in humans are diverse and not conclusive [19,20,21,22,23]. In addition, studies have shown that the dietary glycemic index is directly associated with VC [24], while omega-3 fatty acids and fruits and vegetables intake are inversely related to it [25,26].

The PROGREDIR study is a cohort set in São Paulo, Brazil, comprising essentially participants with CKD classes 3 and 4 who are participating in ongoing follow-up [27]. This study aimed to evaluate cross-sectionally the association between macro- and micronutrient intakes, via a food frequency questionnaire (FFQ) and baseline coronary artery calcification (CAC), in this population.

## 2. Materials and Methods

### 2.1. Study Design and Sample Collection

**Figure A1:**
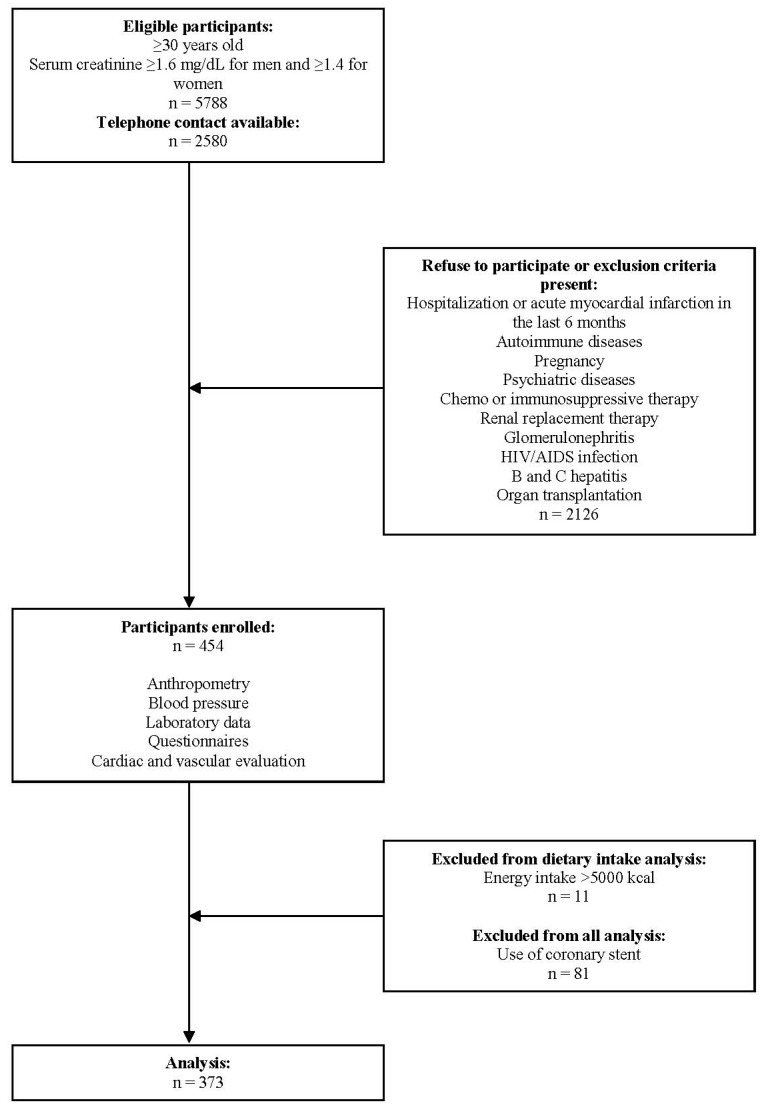
Participant flow chart.

The present study used data from the baseline of the PROGREDIR study. Detailed methods have been published elsewhere [27]. We invited patients from the Hospital das Clínicas Outpatient Service, São Paulo, Brazil, a quaternary care facility, to participate in the study. Initially, patients from outpatient services who were older than 30 years old and had at least two measurements of creatinine (with a minimum interval of 3 months) of ≥1.6 mg/dL for men and ≥1.4 mg/dL for women were eligible. Eligible candidates were contacted by phone and invited to participate in the study, if no exclusion criteria were present (pregnancy, psychiatric diseases, hospitalization or acute myocardial infarction in the last 6 months, ongoing chemo or immunosuppressive therapy, autoimmune diseases, ongoing renal replacement therapy, any organ transplantation, glomerulonephritis, hepatitis B and C, and HIV/AIDS infection) (Figure A1). Data from 454 participants were collected from March 2012 to December 2013. The study was approved by two Ethics Committees (protocol 11147/11, approved on 4 November 2011 and protocol 0798/11, approved on 2 February 2012) and written informed consent was obtained from all participants.

Participants visited the research center for interviews and clinical exams in accordance with standard protocols. Sociodemographic (age, gender and race) and lifestyle (tobacco and alcohol use) variables were self-reported. Hypertension was defined as a previous medical history of the condition and diabetes was defined as a medical history of diabetes, use of medication to treat diabetes, fasting plasma glucose ≥ 126 mg/dL, glycated hemoglobin ≥ 6.5%, or a 2-h plasma glucose ≥ 200 mg/dL (oral glucose tolerance test). Glomerular filtration rate (eGFR) was estimated by the Chronic Kidney Disease Epidemiology Collaboration equation [28]. Previous cardiovascular disease was defined as self-reported history of myocardial infarction or stroke. Current medication was collected by checking the medical prescriptions.

### 2.2. Dietary Intake

We used the validated food frequency questionnaire (FFQ) of Brazilian Longitudinal Study of Adult Health (ELSA Brasil) [29] to evaluate dietary intake. The questionnaire consisted of 114 foods and/or preparations and evaluated the frequency (daily, weekly or monthly) and the usual amount (household measures) of intake of each food/preparation. Furthermore, it also included 19 questions about general characteristics of eating habits, with reference to the last 12 months. FFQ was applied by staff trained in this process. After data collection, we reviewed the FFQ to verify whether the portion sizes of foods were in accordance with what is usually consumed by the Brazilian population.

For the evaluation of energy and nutrient intakes we used the United States Department of Agriculture (USDA) Food Composition Databases [30]. When a USDA nutrient value varied from 80% to 120% in relation to the Brazilian Table of Food Composition [31], we used the value available in the latter. We excluded from analyses patients who had an energy intake higher than 5000 kcal (*n* = 11), because these are unlikely values that could lead to overestimation of nutrient intakes [32]. Macro- and micronutrient intakes were adjusted for energy intake using the residual method [33]. Detailed nutritional data of the cohort has been published elsewhere [34].

### 2.3. Coronary Artery Calcification

CAC was evaluated using the Agatston coronary artery calcium score [35]. Participants underwent a non-contrast computed tomography (CT) scan using a 64 detector CT scanner (Philips Brilliance, Philips, The Netherlands). The field of view was set to include the entire heart, and the *z*-axis direction included data from the bifurcation of the pulmonary arteries to the apex of the heart during an expiratory pause. The default settings included were 120 kV, milliampere adjusted to body mass index (BMI), one phase prospective acquisition at 70% (mid-diastole) of the cardiac cycle and collimation of 2.5 mm, gantry rotation of 400 m/s, and reconstruction with a standard filter. Images were analyzed using the Brilliance Workspace software. The CAC score was calculated using a threshold of 130 Hounsfield CAC was not performed/considered in those with a coronary stent since these devices are known to intensely overestimate CAC measurements [36], leaving 373 participants for the analysis.

### 2.4. Statistical Analysis

Analyses were performed using SPSS software version 17.0 and R version 3.3.1 (generalized linear models and LASSO (least absolute shrinkage and selection operator) regression). Variables were tested for normality using the Kolmogorov–Smirnov test, and then differences between tertiles of CAC were tested using the ANOVA (normal distribution) or Kruskal-Wallis test (non-normal distribution) for continuous variables and the chi-square test for categorical variables. Correlation between continuous variables was evaluated by Pearson’s correlation coefficient for variables with normal distribution and Spearman’s correlation coefficient for variables with non-normal distribution.

For the regression analyses, we used the CAC value plus 0.5 (CAC + 0.5). Sociodemographic, lifestyle and clinical variables related to CAC were evaluated with generalized linear models with gamma distribution. We next evaluated the association between macro- and micronutrient intakes and CAC using generalized linear mixed models with gamma distribution, appropriate for analyzing non-normal data involving random effects [37]. In these models, we used the subject as the random effect, addressing the non-independence between nutrient intakes, and nutrients and confounding variables (age, gender, diabetes mellitus, tobacco use, previous cardiovascular disease and medication use) as fixed effects.

However, the generalized linear model did not allow for adjustments between nutrients, since nutrients significantly related to CAC were very high correlated. Collinearity between nutrients can inflate the variances of the regression coefficients and impair the statistical power [38]. To address this issue, we next evaluated the nutrients mostly related to CAC variability by LASSO regression [39]. LASSO is a method that minimizes the residual sum of squares penalized by the sum of the absolute value of the regression coefficients, which tends to produce some coefficients equal to zero and hence providing the selection of variables [39]. LASSO has several advantages over conventional methods, especially because it allows the selection of variables even when there is collinearity between data [39,40].

## 3. Results

In Table 1, we show descriptive data of the study sample, which was characterized by older patients, predominantly male and had a high percentage of individuals with hypertension (91.4%), diabetes (55.5%) and previous cardiovascular disease (33.0% reporting a previous myocardial infarction or stroke). The median CAC was 165 (IQR 8, 785) and 79.4% participants showed CAC > 0, and 55.2% showed CAC > 100. None of the participants reported being a vegetarian and 56.6% stated that they follow some type of diet (154/41.3% participants using diet for hypertension, 82/22.0% for diabetes and only 5/1.3% for CKD).

Baseline characteristics according to tertiles of CAC are also described in Table 1. The highest tertile of CAC was directly associated with age, male gender, self-reported white race, tobacco use, alcohol use, hypertension, diabetes mellitus, cardiovascular disease, glycated hemoglobin and statin use. Low-density lipoprotein cholesterol (LDL-C) and diastolic blood pressure (DBP) were inversely related to the highest tertile of CAC, possibly as a result of more frequent statin use and older age, respectively. Other medications, such as calcium carbonate, vitamin D, oral hypoglycemic agents and antihypertensive medication were not different among the CAC tertiles. Calcitriol use was very low in the cohort (5.9%). None of the mineral metabolism factors (serum phosphorus, serum total calcium, FGF23, PTH and 25-hydroxyvitamin D) or eGFR or albuminuria were associated with CAC (Table 1).

In the multivariate generalized linear model with gamma distribution that evaluated the clinical variables, those directly associated with CAC were age (β = 0.06, CI 95% 0.04, 0.08, *p* < 0.001), male gender (β = 0.58, CI 95% 0.17, 0.99, *p* = 0.01), diabetes mellitus (β = 0.58, CI 95% 0.19, 0.98, *p* = 0.004), tobacco use (β = 0.87, CI 95% 0.14, 1.59, *p* = 0.02), and cardiovascular disease (β = 0.62, CI 95% 0.21, 1.04, *p* = 0.003), and these were chosen as our main confounding variables.

Table 2 shows macro- and micronutrient intakes according to tertiles of CAC. The highest tertile of CAC was directly associated with intakes of phosphorus, calcium and magnesium, and there was a higher intake of pantothenic acid and potassium among individuals classified in the second tertile (Table 2).

The micronutrients from Table 2 that showed significant associations with CAC were then tested using generalized linear mixed models (Table 3). First, we adjusted for age, gender, diabetes mellitus and tobacco use as fixed effects and subject as a random effect (model 1), and the intakes of pantothenic acid, phosphorus, calcium and potassium remained significantly associated with CAC. These associations were confirmed in a second model (model 2), adjusting now for calcium carbonate use, vitamin D use, statin use and previous cardiovascular disease as fixed effects, in addition to the variables from model 1. We also tested for an interaction between calcium and phosphorus intakes, but no significant association emerged.

**Figure 1:**
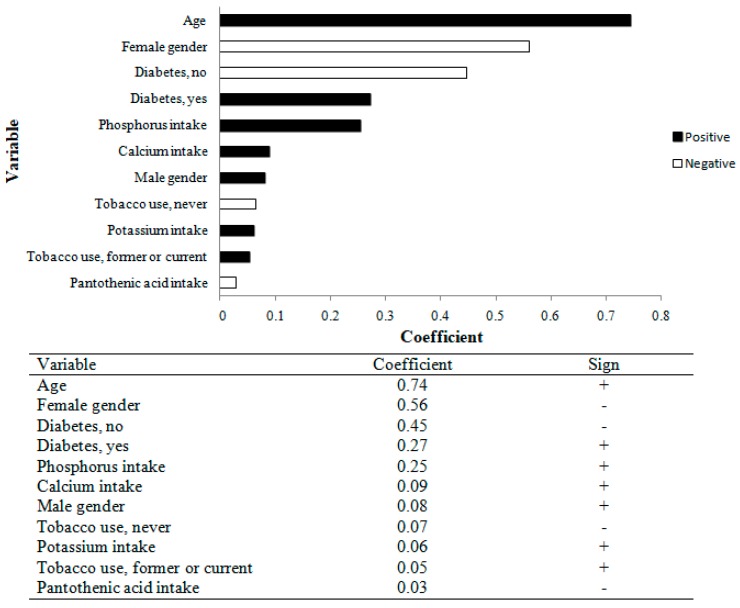
LASSO regression using sociodemographic and clinical variables, and nutrient intake, and CAC among PROGREDIR study participants. CAC, coronary artery calcification.

We next wanted to adjust for micronutrients. However, this would not be appropriate in generalized linear modelling since these nutrients were highly correlated (Table A1). In order to address this issue, we used the LASSO regression to evaluate the nutrients associated with CAC variability. These results are shown in Figure 1, and the nutrients that most explained the variance of CAC were phosphorus, calcium and potassium. We repeated the LASSO regression after excluding individuals with previous cardiovascular disease, and phosphorus (β = 0.15) and potassium (β = 0.12) remained associated with CAC, while calcium was no longer associated with calcification.

## 4. Discussion

In this cross-sectional study, the intakes of phosphorus, calcium and potassium were directly associated with CAC in a CKD population. These three micronutrients remained related to CAC in the LASSO regression, with phosphorus intake having the largest effect on CAC variability. LASSO regression is a biostatistical approach [39] that allows the handling of variables that present collinearity, a violation to assumptions of traditional models. Collinearity is a common issue in diet related data, since nutrients are not consumed in isolation, thus presenting complex correlations, making it difficult to evaluate associations between isolated nutrients and health outcomes [41]. One of the strategies most commonly used to address this issue is the principal component analysis, which transforms correlated variables into a set of non-correlated factors (primary components). However, while correct in terms of statistical analysis, principal component analysis results can be difficult to interpret biologically, particularly when there is interest in testing one specific variable. In this sense, LASSO might be an interesting alternative for selecting variables mostly related to one effect, even when collinearity is present.

The role of a high phosphorus diet in inducing VC has been well established in experimental studies [15,16]. In these studies, a high phosphorus diet was shown to promote calcification. Interestingly, a high phosphorus diet was also related to a reduction in bone strength [42] and signs of cardiac overload and myocardial hypertrophy [15] in CKD models, suggesting that disturbances in mineral metabolism associated with a high phosphorus diet result in a systemic trade-off that is possibly harmful in the long-term.

In vitro, treatment of human vascular smooth muscle cells (VSMC) with high phosphate levels promoted dose-dependent calcification, with upregulation of osteochondrogenic markers, including RUNX2/Cbfa1, Osterix, alkaline phosphatase and osteopontin, and simultaneous downregulation of VSMC markers, such as SM22α and SMMHC [43,44,45]. In addition, it has been demonstrated the role of phosphate in inducing VSMC apoptosis and matrix degradation is associated with mechanisms related to calcification [46]. In humans, in a cross-sectional study conducted in people with normal renal function, there was no association between phosphorus intake and CAC [19]. However, in this single study, phosphorus intakes were lower than the present study.

Calcium intake was also directly associated with CAC in this study. In an in vitro human VSMC model of VC, elevated extracellular calcium levels increased the mineralization of VSMC under normal phosphorus conditions, probably by increasing the product of calcium × phosphorus [47]. Treatment with high calcium and phosphate levels promoted calcification in aortic segments to a greater extent compared to treatment with high phosphorus levels alone [48]. In vivo, treatment with calcium in drinking water promoted a positive calcium balance and calcification [49].

In people with normal kidney function, no association was found between calcium intake and VC, even in populations with a high calcium diet [19,50,51]. On the other hand, in a randomized clinical trial conducted in CKD patients, treatment with calcium-based phosphate binders was related to the progression of VC [20]. According to the K/DOQI (Kidney Disease Outcomes Quality Initiative) [52], the tolerable upper intake level for calcium in CKD population is 2000 mg/day and is the same upper level recommended by DRIs (Dietary Reference Intakes) for the general population [53]. Studies that have evaluated the calcium balance in people with CKD have confirmed this recommendation, demonstrating that a high calcium diet (~2000 mg/day) induces a positive calcium balance, while a calcium intake between 800 and 1000 mg/day promotes a neutral balance [54,55]. Future studies may be conducted in the CKD population to confirm whether a higher calcium intake, even within recommendations, may be associated with CAC as well as the factors involved in this condition.

To our knowledge, an association between potassium intake and VC has not previously been described in CKD. One possibility is that this finding is related to the fact that diets reported in the FFQ may be influenced by the presence of comorbidities and treatments, particularly because our cohort was originally derived from outpatient services of a quaternary hospital. Another hypothesis is that a high potassium intake leads to increases in aldosterone synthesis and secretion [56], which has already been demonstrated as a promoter of calcification [57,58]. Unfortunately, we did not measure aldosterone in our study and could not test if the association found between potassium intake and CAC was mediated by aldosterone. Recently, Sun et al. [59] demonstrated that a reduced potassium intake in ApoE-deficient mice was associated with calcification, presumably though the modulatory effect of serum potassium on intracellular calcium and activation of the vascular smooth muscle cells. However, it is important to note that these findings, which are in the opposite direction of the positive association between potassium intake and CAC found in our analyses, were observed in the setting of normal renal function and low-normal serum values—biological conditions that are very different from those observed in CKD patients.

In experimental and human studies, an inverse association has been shown between a high magnesium intake and calcification [21,60]. In the PROGREDIR study, the median magnesium intake was below the recommended [61] level and this may help explain the lack of association observed. In addition, there is increasing evidence that vitamin K is also a calcification inhibitor, but results in this regard are still controversial. It has been shown that a high vitamin K intake or supplementation is inversely related to calcification in the general population [62,63], but menaquinone (vitamin K2) supplementation does not reduce VC progression in CKD patients [23]. In our study, there was no association between vitamin K intake and CAC.

Our study had some limitations. First, it was a cross-sectional study, and it is therefore not possible to rule out reverse causation as an explanation for some of the associations described, particularly for potassium intake. In addition, the PROGREDIR population is a hospital-derived sample, where diets reported may possibly be influenced by current illnesses and treatments. Second, dietary intake was evaluated by a FFQ, which may not include all foods consumed and may impair the quantification of nutrients [64]. Third, it was not possible to estimate the intake of inorganic phosphate from ultra-processed foods. However, this would presumably lead to an underestimation of phosphorus intake, suggesting that the importance of phosphorus consumption may be even greater than the one observed in our sample. Lastly, all laboratory investigations were performed at a single-time point, a fact that may limit the ability of these measurements to reflect the outcomes studied. Calcitriol use was very low in the cohort, and therefore we could not address the interesting question of whether this drug is associated with calcification among CKD patients.

## 5. Conclusions

We showed a positive association between the intakes of phosphorus, calcium and potassium and CAC in a CKD population through a cross-sectional study. Prospective studies are needed to validate these findings and evaluate the impact of dietetic measures in terms of CAC prevention and progression.

## Figures and Tables

**Table 1:** Baseline characteristics of participants included in the study and according to tertiles of CAC.

Variable ^1^	All *n* = 373	1st Tertile *n* = 124	2nd Tertile *n* = 125	3rd Tertile *n* = 124	*p* ^2^
CAC score (min, max)		(0, 44)	(45, 513)	(514, 10,357)	
Age, years	68 (60, 77)	62 (52, 73)	69 (59, 77)	73 (65, 78)	<0.001
Male gender, *n* (%)	232 (62.2)	66 (53.2)	75 (60.0)	91 (73.4)	0.004
White race, *n* (%)	225 (60.3)	60 (48.4)	77 (61.6)	88 (71.0)	0.001
Former/current tobacco use, *n* (%)	212 (56.8)	59 (47.6)	72 (57.6)	81 (65.3)	0.02
Former/current alcohol use, *n* (%)	248 (66.5)	76 (61.3)	78 (62.4)	94 (75.8)	0.03
Hypertension, *n* (%)	341 (91.4)	107 (86.3)	117 (93.6)	117 (94.4)	0.05
Diabetes, *n* (%)	207 (55.5)	55 (44.4)	69 (55.2)	83 (66.9)	0.002
Cardiovascular disease, *n* (%)	123 (33.0)	23 (18.5)	35 (28.0)	65 (52.4)	<0.001
BMI, kg/m^2^	29 (26, 32)	29 (25, 32)	29 (26, 33)	28 (26, 31)	0.46
eGFR, mL/min/1.73 m^2^	37.5 ± 14.8	37.7 ± 15.3	38.2 ± 14.4	36.7 ± 14.7	0.72
Albuminuria, mg/g creatinine	92 (16, 667)	130 (16, 736)	88 (17, 730)	70 (16, 549)	0.81
FGF23, RU/mL	95 (70, 130)	91 (67, 123)	92 (76, 126)	106 (69, 139)	0.23
PTH, pg/mL	94 (65, 143)	88 (65, 145)	103 (66, 151)	94 (64, 139)	0.91
25-hydroxyvitamin D, ng/mL	25 ± 11	26 ± 10	24 ± 9	26 ± 12	0.57
Serum phosphorus, mg/dL	3.7 ± 0.6	3.6 ± 0.7	3.7 ± 0.7	3.6 ± 0.6	0.46
Serum total calcium, mg/dL	9.6 (9.2, 9.9)	9.6 (9.3, 9.9)	9.5 (9.2, 9.9)	9.6 (9.2, 9.9)	0.88
Serum potassium, mEq/L	4.6 ± 0.5	4.6 ± 0.6	4.6 ± 0.5	4.6 ± 0.5	0.55
Glycated hemoglobin, %	6.2 (5.8, 7.1)	6.0 (5.7, 6.5)	6.2 (5.9, 7.2)	6.4 (5.9, 7.7)	0.001
LDL-C, mg/dL	89 (70, 112)	92 (72, 115)	94 (75, 122)	80 (63, 100)	0.001
Triglycerides, mg/dL	140 (99, 193)	137 (99, 182)	147 (102, 191)	139 (94, 202)	0.60
SBP, mmHg	140 ± 24	137 ± 24	143 ± 23	141 ± 24	0.17
DBP, mmHg	75 (69, 84)	76 (71, 85)	76 (69, 85)	72 (64, 83)	0.01
Calcium carbonate use, *n* (%)	28 (7.5)	11 (8.9)	8 (6.4)	9 (7.3)	0.72
Vitamin D use, *n* (%)	92 (24.7)	35 (28.2)	27 (21.6)	30 (24.2)	0.41
Oral hypoglycemic agents use, *n* (%)	92 (24.7)	23 (18.5)	36 (28.8)	33 (26.6)	0.14
Insulin use, *n* (%)	82 (22.0)	19 (15.3)	31 (24.8)	32 (25.8)	0.12
Statin use, *n* (%)	221 (59.2)	63 (50.8)	72 (57.6)	86 (69.4)	0.03
Antihypertensive medication use, *n* (%)	340 (91.2)	110 (88.7)	112 (89.6)	118 (95.2)	0.15
VKA use, *n* (%)	32 (8.6)	10 (8.1)	9 (7.2)	13 (10.5)	0.71
Follow some diet, *n* (%)	211 (56.6)	70 (56.5)	69 (55.2)	72 (58.1)	0.90

BMI, body mass index; CAC, coronary artery calcification; DBP, diastolic blood pressure; eGFR, estimated glomerular filtration rate; FGF23, fibroblast growth factor 23; LDL-C, low-density lipoprotein cholesterol; PTH, parathyroid hormone; SBP, systolic blood pressure, VKA, vitamin K antagonists. ^1^ number (percentage), mean ± standard deviation or median (interquartile range). ^2^ *p*-trend for comparison between tertiles of CAC.

**Table 2:** Nutrient intakes of participants included in the study according to tertiles of CAC.

Nutrient ^1^	1st Tertile *n* = 124	2nd Tertile *n* = 125	3rd Tertile *n* = 124	*p* ^2^
CAC score (min, max)	(0, 44)	(45, 513)	(514, 10,357)	
Carbohydrate, g	288 ± 43	294 ± 39	285 ± 40	0.25
Protein, g	83 (72, 98)	80 (69, 97)	84 (78, 104)	0.77
Total fat, g	50 ± 11	49 ± 10	52 ± 12	0.13
Saturated fat, g	17.1 (13.2, 21.3)	16.7 (13.4, 21.2)	17.1 (13.3, 19.1)	0.55
Monounsaturated fat, g	17.3 ± 4.4	16.4 ± 4.1	17.8 ± 5.7	0.06
Polyunsaturated fat, g	15.3 ± 3.4	14.8 ± 3.5	15.3 ± 3.6	0.48
Omega-3 fatty acids, g	2.2 (2.0, 2.6)	2.3 (1.9, 2.7)	2.3 (1.9, 2.7)	0.82
Thiamine, mg	1.2 (1.0, 1.6)	1.4 (1.0, 1.8)	1.2 (1.0, 2.0)	0.15
Riboflavin, mg	1.18 (0.83, 1.56)	1.34 (0.97, 1.80)	1.32 (0.78, 1.62)	0.08
Niacin, mg	20 (15-29)	21 (15–34)	21 (19–35)	0.61
Pantothenic acid, mg	5.82 (5.23, 6.38)	6.04 (5.47, 6.84)	5.88 (5.62, 7.49)	0.05
Pyridoxine, mg	0.7 (0.5, 0.9)	0.7 (0.5, 0.9)	0.7 (0.5, 0.8)	0.59
Folate, µg	504 (446, 589)	525 (451, 618)	532 (433, 620)	0.33
Cobalamin, µg	3.4 (2.5, 4.8)	3.7 (2.6, 5.1)	3.9 (3.1, 5.6)	0.35
Vitamin K, µg	159 (100, 229)	154 (95, 275)	162 (149, 292)	0.80
Phosphorus, mg	1138 ± 222	1193 ± 270	1212 ± 209	0.04
Calcium, mg	688 (500, 871)	740 (536, 957)	792 (420, 916)	0.01
Zinc, mg	10.1 (8.4, 12.3)	9.5 (8.2, 11.2)	10.2 (9.3, 12.7)	0.14
Magnesium, mg	259 (235, 306)	287 (245, 332)	289 (235, 341)	0.01
Potassium, mg	2892 ± 626	3141 ± 819	3082 ± 622	0.02
Sodium, mg	2149 (1838, 2488)	2164 (1859, 2445)	2285 (1828, 2802)	0.30
Selenium, µg	122 (104, 142)	118 (100, 137)	121 (111, 150)	0.59

CAC, coronary artery calcification. ^1^ mean ± standard deviation or median (interquartile range). ^2^ *p*-trend for comparison between tertiles of CAC.

**Table 3:** Generalized linear mixed models between nutrient intakes and CAC among participants included in the study.

Nutrient ^1^	β	CI 95%	*p*
*Model 1—Nutrients adjusted for age, gender, diabetes mellitus and tobacco use as fixed effects and subject as a random effect*			
Pantothenic acid	0.48	0.22, 0.75	<0.001
Phosphorus	0.38	0.10, 0.65	0.01
Calcium	0.0008	0.0001, 0.0017	0.04
Potassium	0.0005	0.0001, 0.0010	0.02
Monounsaturated fat	0.03	−0.03, 0.09	0.34
Riboflavin	0.20	−0.09, 0.49	0.17
Magnesium	0.001	−0.001, 0.003	0.28
*Model 2—Nutrients adjusted for age, gender, diabetes mellitus, tobacco use, calcium carbonate use, vitamin D use, statin use and cardiovascular disease as fixed effects and subject as a random effect*			
Pantothenic acid	0.40	0.11, 0.70	0.01
Phosphorus	0.43	0.14, 0.72	0.004
Calcium	0.28	−0.01, 0.57	0.06
Potassium	0.45	0.18, 0.73	0.001

CAC, coronary artery calcification; CI, confidence interval. Dependent variable: CAC + 0.5. ^1^ Nutrients were standardized [(X_i_ − mean)/standard deviation].

## Appendix B

**Table A1:** Correlation between nutrient intakes of participants from the PROGREDIR study.

Nutrient	Pantothenic Acid, mg		Phosphorus, mg		Calcium, mg		Potassium, mg	
	*r*	*p*	*r*	*p*	*r*	*p*	*r*	*p*
Pantothenic acid, mg	1.00	-	0.42	<0.001	0.36	<0.001	0.53	<0.001
Phosphorus, mg	0.42	<0.001	1.00	-	0.59	<0.001	0.44	<0.001
Calcium, mg	0.36	<0.001	0.59	<0.001	1.00	-	0.29	<0.001
Potassium, mg	0.53	<0.001	0.44	<0.001	0.29	<0.001	1.00	-

*r*, correlation coefficient.
